# (*E*)-13-(2-Bromo­phen­yl)micheliolide

**DOI:** 10.1107/S1600536814002177

**Published:** 2014-02-05

**Authors:** Narsimha Reddy Penthala, Shobanbabu Bommagani, Venumadhav Janganati, Sean Parkin, Peter A. Crooks

**Affiliations:** a Department of Pharmaceutical Sciences, College of Pharmacy, University of Arkansas for Medical Sciences, Little Rock, AR 72205, USA; b Department of Chemistry, University of Kentucky, Lexington, KY 40506, USA

## Abstract

The title compound, C_21_H_23_BrO_3_ [systematic name: (3*E*,3a*S*,6*Z*,9*R*,9a*S*,9b*S*)-3-(2-bromo­benzyl­idene)-9-hy­droxy-6,9-dimethyl-3,3a,4,5,7,8,9,9a-octa­hydro­azuleno[4,5-*b*]furan-2(9b*H*)-one] was prepared by the reaction of 1-bromo-2-iodo­benzene with micheliolide [systematic name: (3a*S*,*R*,9a*S*,9b*S*,*Z*)-9-hy­droxy-6,9-dimethyl-3-methyl­ene-3,3a,4,5,7,8,9,9a-octa­hydro­azuleno[4,5-*b*]furan-2(9*bH*)-one] under Heck reaction conditions. The title compound exhibits intra­molecular O—H⋯O hydrogen bonding between the hy­droxy group and the lactone ring O atom, forming a ring of graph-set motif *S*(6). The 2-bromo­phenyl group is *trans* to the lactone ring, indicating that this is the *E* isomer (geometry of the exocyclic C=C bond). The dihedral angle between the benzene ring of the 2-bromo­phenyl moiety and the mean plane of the lactone ring is 51.68 (7)°.

## Related literature   

For the biological activity of micheliolide Michael addition compounds, see: Rodriguez *et al.* (1976 ▶); Sethi *et al.* (1984 ▶); Neelakantan *et al.* (2009 ▶); Zhang *et al.* (2012 ▶). For details of the Heck chemistry, see: Han *et al.* (2009 ▶). For the crystal structure of micheliolide, see: Acosta *et al.* (1991 ▶). For the crystal structure of a similar compound, see: Penthala *et al.* (2013 ▶).
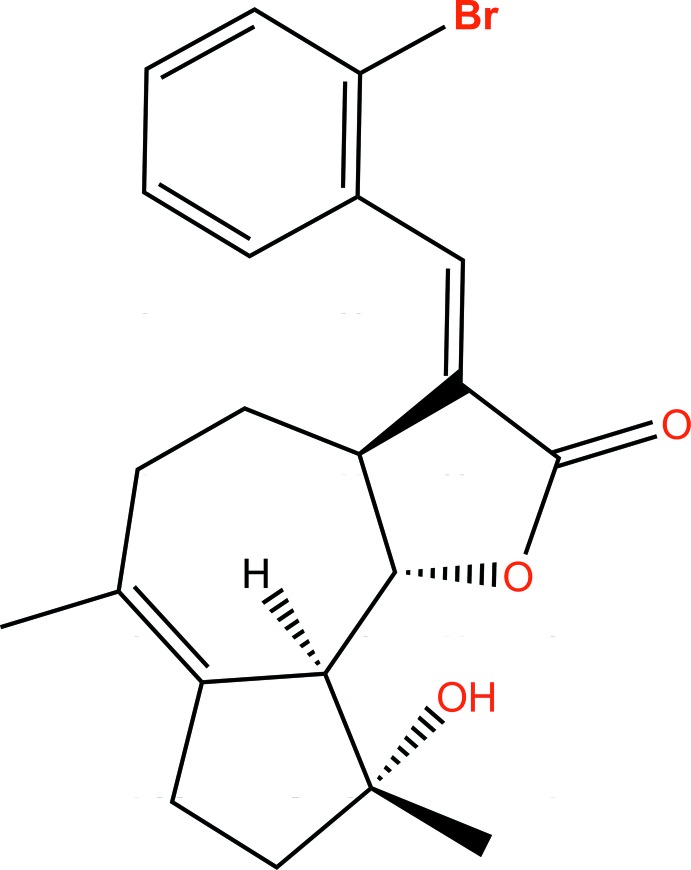


## Experimental   

### 

#### Crystal data  

C_21_H_23_BrO_3_
*M* *_r_* = 403.30
Orthorhombic, 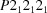

*a* = 7.1617 (1) Å
*b* = 13.1615 (2) Å
*c* = 19.2306 (3) Å
*V* = 1812.65 (5) Å^3^
*Z* = 4
Mo *K*α radiation
μ = 2.29 mm^−1^
*T* = 90 K
0.20 × 0.20 × 0.15 mm

#### Data collection  

Nonius KappaCCD diffractometer
Absorption correction: multi-scan (*SADABS*; Sheldrick, 2008*a* ▶) *T*_min_ = 0.550, *T*_max_ = 0.714
48139 measured reflections
4148 independent reflections
3871 reflections with *I* > 2σ(*I*)
*R*_int_ = 0.047

#### Refinement  

*R*[*F* ^2^ > 2σ(*F* ^2^)] = 0.025
*wR*(*F* ^2^) = 0.064
*S* = 1.04
4148 reflections
231 parameters
H atoms treated by a mixture of independent and constrained refinement
Δρ_max_ = 0.41 e Å^−3^
Δρ_min_ = −0.34 e Å^−3^
Absolute structure: Flack parameter determined using 1569 quotients [(*I* ^+^)−(*I* ^−^)]/[(*I* ^+^)+(*I* ^−^)] (Parsons *et al.*, 2013 ▶)
Absolute structure parameter: 0.032 (3)

### 

Data collection: *COLLECT* (Nonius, 1998 ▶); cell refinement: *SCALEPACK* (Otwinowski & Minor, 2006 ▶); data reduction: *DENZO-SMN* (Otwinowski & Minor, 2006 ▶); program(s) used to solve structure: *SHELXS97* (Sheldrick, 2008*b* ▶); program(s) used to refine structure: *SHELXL2013* (Sheldrick, 2008*b* ▶); molecular graphics: *XP in *SHELXTL** (Sheldrick, 2008*b* ▶); software used to prepare material for publication: *SHELXL97* (Sheldrick, 2008*b* ▶).

## Supplementary Material

Crystal structure: contains datablock(s) global, I. DOI: 10.1107/S1600536814002177/sj5372sup1.cif

Structure factors: contains datablock(s) I. DOI: 10.1107/S1600536814002177/sj5372Isup2.hkl

Supporting information file. DOI: 10.1107/S1600536814002177/sj5372Isup3.cml

CCDC reference:

Additional supporting information:  crystallographic information; 3D view; checkCIF report

## Figures and Tables

**Table 1:** Hydrogen-bond geometry (Å, °)

*D*—H⋯*A*	*D*—H	H⋯*A*	*D*⋯*A*	*D*—H⋯*A*
O3—H3⋯O2	0.77 (4)	2.26 (4)	2.883 (3)	139 (4)

**Fig. 1.**
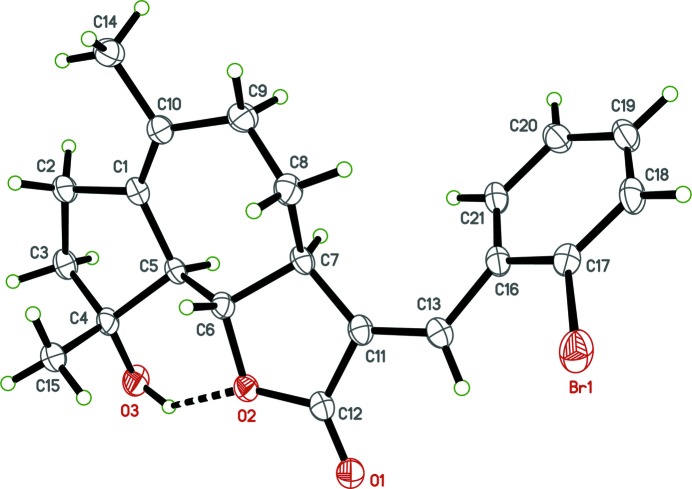
A view of the molecule with displacement ellipsoids drawn at the 50% probability level.

## supplementary crystallographic information

### 1. Comment

Micheliolide (MCL) belongs to the class of guaianolide sesquiterpene lactones, which are being developed for the treatment of cancer (Sethi *et al.*, 1984 and Zhang *et al.*, 2012). The exocyclic double bond in such sesquiterpenes is believed to be responsible for their biological properties because of its exceptional chemical reactivity with nucleophilic groups (Rodriguez *et al.*, 1976). The MCL crystal structure was described by Acosta *et al.* (1991). Recently, micheliolide Michael addition analogs were reported as potent anti-leukemic agents (Zhang *et al.*, 2012). In a recent communication we reported the crystal structure of (*E*)-13-(4-aminophenyl)parthenolide, a Heck reaction derivative of parthenolide (Penthala *et al.*, 2013). Now, in continuation of our research on sesquiterpene lactones as anti-leukemic agents (Neelakantan *et al.*, 2009), we are focusing on the synthesis of the title *E*-olefinic analogue of micheliolide, which was obtained from the reaction of micheliolide with 1-bromo-2-iodobenzene utilizing Heck chemistry (Han *et al.*, 2009). In order to obtain detailed information on the structural conformation of the title compound and to establish the geometry of the exocyclic double bond, a single-crystal X-ray structure determination has been carried out.

Recrystallization of the title compound from hexanes gave colorless needles that were suitable for X-ray analysis. The title molecule, Fig. 1, contains a central seven-membered carbocyclic ring fused to a 5-membered carbocylic ring and a *trans* lactone ring. The two five-membered rings are in half-chair conformations, as reported in the literature for the parent compound, micheliolide (Acosta *et al.*, 1991). The X-ray studies revealed that the title compound exhibits intramolecular O—H···O hydrogen bonding to form a ring of graph-set motif *S*(6). The 2-bromophenyl group is oriented *trans* to the lactone ring to form the *E* isomer (geometry of the exocyclic C═C bond). The H atom of the hydroxy group in the molecule forms an intramolecular hydrogen bond with the lactone ring oxygen atom. The dihedral angle between the benzene ring of the 2-bromophenyl group and the mean plane of the lactone ring is 51.68 (7)°.

### 2. Experimental

A mixture of micheliolide (prepared from parthenolide, Zhang *et al.*, 2012) (50 mg, 0.20 mmol), triethylamine (60 mg, 0.61 mmol), and 1-bromo-2-iodobenzene (63 mg, 0.22 mmol) in dimethylformamide (1 ml) was treated with palladium(II) acetate (0.5 mg, 0.002 mmol) and then stirred at 353 K for 24 h. The reaction mixture was cooled to room temperature, water (8 ml) was added, and the mixture was extracted with ethyl acetate (10 ml x 3). The separated organics were dried over anhydrous Na_2_SO_4_ and concentrated under reduced pressure. The crude residue was purified using silica flash chromatography (7:3, hexanes/EtOAc) to afford the title compound, which was recrystallized from hexanes as colorless needles suitable for X-ray analysis (65 mg, 80% yield; M·P: 421–423 K); ^1^H NMR (400 MHz, CDCl_3_): δ 7.70–7.70 (d, *J*=2.8 Hz, 1H), 7.62–7.64 (d, *J*=8.0 Hz, 1H), 7.22–7.35 (m, 3H, Ar—H and =CH), 3.89–3.94 (t, *J*=10 Hz, 20.4 Hz, 1H), 3.01–3.06 (t, *J*=8.8 Hz, 19.6 Hz, 1H), 2.73–2.76 (d, *J*=11.2 Hz, 1H), 2.71 (s, 1H), 2.35–2.41 (m, 1H), 2.01–2.21 (m, 1H), 1.76–1.89 (m, 3H), 1.61 (s, 3H), 1.32 (s, 3H), 1.00–1.04 (m, 1H) *p.p.m.*. ^13^C NMR (100 MHz, CDCl_3_): δ 22.57, 23.69, 24.91, 29.86, 35.36, 38.23, 48.93, 58.99, 80.79, 124.14, 126.93, 129.86, 130.47, 130.55, 131.03, 131.50, 132.86, 134.54, 136.70, 170.68 *p.p.m.*.

### 3. Refinement

H atoms were found in difference Fourier maps. Carbon-bound H atoms were subsequently placed at idealized positions with constrained distances of 0.98 Å (RCH_3_), 0.99 Å (*R*_2_CH_2_), 1.00 Å (*R*_3_CH) and 0.95 Å (C_sp2_H). The hydroxy hydrogen coordinates were refined. *U*_iso_(H) values were set to either 1.2*U*_eq_ or 1.5*U*_eq_ (RCH_3_, OH) of the attached atom.

### Crystal data

**Table:**

C_21_H_23_BrO_3_	*D*_x_ = 1.478 Mg m^−^^3^
*M**_r_* = 403.30	Mo *K*α radiation, λ = 0.71073 Å
Orthorhombic, *P*2_1_2_1_2_1_	Cell parameters from 127083 reflections
*a* = 7.1617 (1) Å	θ = 1.0–27.5°
*b* = 13.1615 (2) Å	µ = 2.29 mm^−^^1^
*c* = 19.2306 (3) Å	*T* = 90 K
*V* = 1812.65 (5) Å^3^	Block, cut from needle, colourless
*Z* = 4	0.20 × 0.20 × 0.15 mm
*F*(000) = 832	

### Data collection

**Table:**

Nonius KappaCCD diffractometer	4148 independent reflections
Radiation source: fine-focus sealed-tube	3871 reflections with *I* > 2σ(*I*)
Detector resolution: 9.1 pixels mm^-1^	*R*_int_ = 0.047
φ and ω scans at fixed χ = 55°	θ_max_ = 27.5°, θ_min_ = 1.9°
Absorption correction: multi-scan (*SADABS*; Sheldrick, 2008*a*)	*h* = −9→9
*T*_min_ = 0.550, *T*_max_ = 0.714	*k* = −17→17
48139 measured reflections	*l* = −24→24

### Refinement

**Table:**

Refinement on *F*^2^	Secondary atom site location: difference Fourier map
Least-squares matrix: full	Hydrogen site location: mixed
*R*[*F*^2^ > 2σ(*F*^2^)] = 0.025	H atoms treated by a mixture of independent and constrained refinement
*wR*(*F*^2^) = 0.064	*w* = 1/[σ^2^(*F*_o_^2^) + (0.0352*P*)^2^ + 0.4253*P*] where *P* = (*F*_o_^2^ + 2*F*_c_^2^)/3
*S* = 1.04	(Δ/σ)_max_ = 0.001
4148 reflections	Δρ_max_ = 0.41 e Å^−^^3^
231 parameters	Δρ_min_ = −0.34 e Å^−^^3^
0 restraints	Absolute structure: Flack parameter determined using 1569 quotients [(*I*^+^)-(*I*^-^)]/[(*I*^+^)+(*I*^-^)] (Parsons *et al.*, 2013)
Primary atom site location: structure-invariant direct methods	Absolute structure parameter: 0.032 (3)

### Special details

**Table:**

Experimental. The crystal was mounted with polyisobutene oil on the tip of a fine glass fibre, fastened in a copper mounting pin with electrical solder. It was placed directly into the cold stream of a liquid nitrogen based cryostat.Diffraction data were collected with the crystal at 90 K, which is standard practice in this laboratory for the majority of flash-cooled crystals.
Geometry. All e.s.d.'s (except the e.s.d. in the dihedral angle between two l.s. planes) are estimated using the full covariance matrix. The cell e.s.d.'s are taken into account individually in the estimation of e.s.d.'s in distances, angles and torsion angles; correlations between e.s.d.'s in cell parameters are only used when they are defined by crystal symmetry. An approximate (isotropic) treatment of cell e.s.d.'s is used for estimating e.s.d.'s involving l.s. planes.

### Fractional atomic coordinates and isotropic or equivalent isotropic displacement parameters (Å^2^)

**Table:**

	*x*	*y*	*z*	*U*_iso_*/*U*_eq_	
Br1	0.79983 (4)	−0.11654 (2)	0.35920 (2)	0.03215 (9)	
O1	0.7715 (3)	0.26157 (14)	0.45446 (10)	0.0248 (4)	
O2	0.5251 (3)	0.34175 (14)	0.40803 (9)	0.0215 (4)	
O3	0.3499 (3)	0.53339 (15)	0.43985 (11)	0.0285 (5)	
H3	0.434 (6)	0.498 (3)	0.444 (2)	0.043*	
C1	0.0338 (3)	0.3743 (2)	0.33548 (13)	0.0202 (5)	
C2	−0.0575 (4)	0.4794 (2)	0.33076 (16)	0.0252 (6)	
H2A	−0.0553	0.5046	0.2823	0.030*	
H2B	−0.1887	0.4765	0.3468	0.030*	
C3	0.0577 (4)	0.5486 (2)	0.37792 (14)	0.0243 (6)	
H3A	0.0633	0.6184	0.3589	0.029*	
H3B	0.0037	0.5512	0.4253	0.029*	
C4	0.2513 (4)	0.50008 (19)	0.37900 (13)	0.0208 (5)	
C5	0.2022 (3)	0.38520 (18)	0.38337 (12)	0.0185 (4)	
H5	0.1602	0.3704	0.4319	0.022*	
C6	0.3602 (3)	0.31433 (18)	0.36651 (14)	0.0192 (5)	
H6	0.3917	0.3211	0.3161	0.023*	
C7	0.3212 (4)	0.20208 (18)	0.38266 (13)	0.0198 (5)	
H7	0.2314	0.1984	0.4224	0.024*	
C8	0.2325 (4)	0.1511 (2)	0.31966 (14)	0.0237 (6)	
H8A	0.2408	0.0764	0.3251	0.028*	
H8B	0.3031	0.1702	0.2774	0.028*	
C9	0.0275 (4)	0.1814 (2)	0.31045 (16)	0.0259 (6)	
H9A	−0.0413	0.1538	0.3509	0.031*	
H9B	−0.0193	0.1449	0.2690	0.031*	
C10	−0.0321 (4)	0.2927 (2)	0.30252 (14)	0.0212 (5)	
C11	0.5102 (4)	0.1654 (2)	0.40740 (13)	0.0201 (5)	
C12	0.6223 (4)	0.2566 (2)	0.42595 (13)	0.0204 (5)	
C13	0.5791 (4)	0.07309 (19)	0.42009 (13)	0.0210 (5)	
H13	0.7092	0.0697	0.4295	0.025*	
C14	−0.2025 (4)	0.3016 (2)	0.25629 (14)	0.0257 (5)	
H14A	−0.2471	0.3720	0.2565	0.039*	
H14B	−0.3011	0.2567	0.2738	0.039*	
H14C	−0.1695	0.2817	0.2087	0.039*	
C15	0.3630 (4)	0.5270 (2)	0.31404 (15)	0.0255 (6)	
H15A	0.3776	0.6010	0.3111	0.038*	
H15B	0.2969	0.5024	0.2727	0.038*	
H15C	0.4864	0.4951	0.3165	0.038*	
C16	0.4774 (4)	−0.0247 (2)	0.42131 (13)	0.0204 (5)	
C17	0.5616 (4)	−0.1163 (2)	0.40254 (13)	0.0220 (5)	
C18	0.4756 (4)	−0.2095 (2)	0.41364 (14)	0.0252 (6)	
H18	0.5356	−0.2707	0.3999	0.030*	
C19	0.3015 (5)	−0.21243 (19)	0.44500 (14)	0.0261 (6)	
H19	0.2434	−0.2759	0.4542	0.031*	
C20	0.2116 (4)	−0.1224 (2)	0.46308 (13)	0.0251 (5)	
H20	0.0909	−0.1244	0.4835	0.030*	
C21	0.2988 (4)	−0.02945 (19)	0.45117 (13)	0.0228 (5)	
H21	0.2364	0.0317	0.4634	0.027*	

### Atomic displacement parameters (Å^2^)

**Table:**

	*U*^11^	*U*^22^	*U*^33^	*U*^12^	*U*^13^	*U*^23^
Br1	0.03169 (14)	0.02821 (14)	0.03656 (15)	0.00934 (12)	0.00802 (12)	0.00322 (13)
O1	0.0264 (10)	0.0225 (9)	0.0254 (9)	−0.0003 (8)	−0.0026 (8)	0.0020 (8)
O2	0.0249 (9)	0.0140 (8)	0.0256 (9)	−0.0014 (7)	−0.0045 (7)	0.0001 (7)
O3	0.0383 (12)	0.0152 (9)	0.0319 (11)	0.0016 (8)	−0.0109 (9)	−0.0040 (8)
C1	0.0231 (11)	0.0163 (12)	0.0212 (11)	−0.0009 (10)	0.0027 (9)	0.0025 (10)
C2	0.0257 (13)	0.0145 (12)	0.0355 (15)	0.0004 (10)	−0.0034 (11)	0.0028 (11)
C3	0.0301 (13)	0.0148 (12)	0.0281 (14)	0.0020 (10)	0.0017 (11)	0.0004 (10)
C4	0.0289 (12)	0.0112 (11)	0.0224 (13)	−0.0009 (9)	−0.0046 (10)	−0.0001 (9)
C5	0.0231 (11)	0.0120 (10)	0.0204 (10)	−0.0016 (12)	0.0008 (9)	0.0005 (9)
C6	0.0242 (11)	0.0148 (11)	0.0185 (12)	−0.0012 (9)	−0.0028 (10)	−0.0006 (10)
C7	0.0258 (12)	0.0119 (11)	0.0218 (12)	−0.0010 (10)	0.0007 (10)	−0.0009 (9)
C8	0.0304 (14)	0.0162 (12)	0.0246 (13)	0.0016 (10)	−0.0023 (11)	−0.0038 (10)
C9	0.0290 (13)	0.0172 (13)	0.0316 (14)	0.0001 (11)	−0.0044 (11)	−0.0055 (11)
C10	0.0229 (12)	0.0187 (13)	0.0221 (13)	0.0003 (10)	0.0010 (10)	0.0000 (10)
C11	0.0261 (13)	0.0175 (13)	0.0168 (12)	0.0002 (10)	0.0008 (10)	0.0004 (10)
C12	0.0254 (13)	0.0176 (12)	0.0182 (12)	−0.0011 (10)	0.0006 (10)	0.0013 (10)
C13	0.0255 (13)	0.0184 (12)	0.0191 (12)	−0.0010 (10)	0.0022 (10)	0.0006 (10)
C14	0.0236 (12)	0.0222 (13)	0.0312 (14)	−0.0014 (12)	−0.0005 (12)	−0.0016 (11)
C15	0.0262 (13)	0.0179 (13)	0.0323 (15)	−0.0013 (10)	−0.0030 (11)	0.0043 (11)
C16	0.0277 (13)	0.0157 (12)	0.0179 (12)	0.0002 (10)	−0.0028 (11)	0.0009 (10)
C17	0.0243 (12)	0.0205 (12)	0.0211 (12)	0.0033 (12)	−0.0017 (9)	0.0009 (11)
C18	0.0360 (15)	0.0148 (13)	0.0247 (14)	0.0045 (11)	−0.0032 (12)	−0.0021 (11)
C19	0.0361 (14)	0.0163 (12)	0.0259 (13)	−0.0040 (13)	−0.0051 (14)	0.0014 (10)
C20	0.0275 (12)	0.0214 (12)	0.0264 (12)	−0.0042 (13)	−0.0001 (11)	0.0025 (11)
C21	0.0279 (12)	0.0155 (12)	0.0251 (12)	0.0024 (12)	−0.0004 (13)	0.0019 (10)

### Geometric parameters (Å, º)

**Table:**

Br1—C17	1.899 (3)	C8—H8B	0.9900
O1—C12	1.203 (3)	C9—C10	1.533 (4)
O2—C12	1.364 (3)	C9—H9A	0.9900
O2—C6	1.470 (3)	C9—H9B	0.9900
O3—C4	1.435 (3)	C10—C14	1.514 (4)
O3—H3	0.77 (4)	C11—C13	1.335 (4)
C1—C10	1.333 (4)	C11—C12	1.487 (4)
C1—C5	1.525 (3)	C13—C16	1.479 (4)
C1—C2	1.532 (4)	C13—H13	0.9500
C2—C3	1.527 (4)	C14—H14A	0.9800
C2—H2A	0.9900	C14—H14B	0.9800
C2—H2B	0.9900	C14—H14C	0.9800
C3—C4	1.527 (4)	C15—H15A	0.9800
C3—H3A	0.9900	C15—H15B	0.9800
C3—H3B	0.9900	C15—H15C	0.9800
C4—C15	1.525 (4)	C16—C17	1.396 (4)
C4—C5	1.555 (3)	C16—C21	1.403 (4)
C5—C6	1.502 (3)	C17—C18	1.389 (4)
C5—H5	1.0000	C18—C19	1.386 (4)
C6—C7	1.535 (3)	C18—H18	0.9500
C6—H6	1.0000	C19—C20	1.393 (4)
C7—C11	1.514 (4)	C19—H19	0.9500
C7—C8	1.524 (4)	C20—C21	1.392 (4)
C7—H7	1.0000	C20—H20	0.9500
C8—C9	1.532 (4)	C21—H21	0.9500
C8—H8A	0.9900		
			
C12—O2—C6	110.22 (19)	C8—C9—H9A	106.9
C4—O3—H3	106 (3)	C10—C9—H9A	106.9
C10—C1—C5	130.0 (2)	C8—C9—H9B	106.9
C10—C1—C2	123.2 (2)	C10—C9—H9B	106.9
C5—C1—C2	106.8 (2)	H9A—C9—H9B	106.7
C3—C2—C1	105.8 (2)	C1—C10—C14	120.2 (2)
C3—C2—H2A	110.6	C1—C10—C9	128.6 (2)
C1—C2—H2A	110.6	C14—C10—C9	110.8 (2)
C3—C2—H2B	110.6	C13—C11—C12	119.4 (2)
C1—C2—H2B	110.6	C13—C11—C7	132.7 (2)
H2A—C2—H2B	108.7	C12—C11—C7	107.5 (2)
C4—C3—C2	104.4 (2)	O1—C12—O2	121.6 (2)
C4—C3—H3A	110.9	O1—C12—C11	129.3 (2)
C2—C3—H3A	110.9	O2—C12—C11	109.1 (2)
C4—C3—H3B	110.9	C11—C13—C16	127.8 (2)
C2—C3—H3B	110.9	C11—C13—H13	116.1
H3A—C3—H3B	108.9	C16—C13—H13	116.1
O3—C4—C15	109.8 (2)	C10—C14—H14A	109.5
O3—C4—C3	109.3 (2)	C10—C14—H14B	109.5
C15—C4—C3	111.6 (2)	H14A—C14—H14B	109.5
O3—C4—C5	111.4 (2)	C10—C14—H14C	109.5
C15—C4—C5	112.9 (2)	H14A—C14—H14C	109.5
C3—C4—C5	101.7 (2)	H14B—C14—H14C	109.5
C6—C5—C1	114.0 (2)	C4—C15—H15A	109.5
C6—C5—C4	115.0 (2)	C4—C15—H15B	109.5
C1—C5—C4	103.7 (2)	H15A—C15—H15B	109.5
C6—C5—H5	107.9	C4—C15—H15C	109.5
C1—C5—H5	107.9	H15A—C15—H15C	109.5
C4—C5—H5	107.9	H15B—C15—H15C	109.5
O2—C6—C5	109.59 (19)	C17—C16—C21	117.4 (2)
O2—C6—C7	105.82 (19)	C17—C16—C13	122.3 (2)
C5—C6—C7	114.6 (2)	C21—C16—C13	119.7 (2)
O2—C6—H6	108.9	C18—C17—C16	122.1 (2)
C5—C6—H6	108.9	C18—C17—Br1	117.7 (2)
C7—C6—H6	108.9	C16—C17—Br1	120.2 (2)
C11—C7—C8	118.8 (2)	C19—C18—C17	119.3 (3)
C11—C7—C6	102.0 (2)	C19—C18—H18	120.3
C8—C7—C6	109.8 (2)	C17—C18—H18	120.3
C11—C7—H7	108.6	C18—C19—C20	120.1 (2)
C8—C7—H7	108.6	C18—C19—H19	120.0
C6—C7—H7	108.6	C20—C19—H19	120.0
C7—C8—C9	112.1 (2)	C21—C20—C19	119.9 (2)
C7—C8—H8A	109.2	C21—C20—H20	120.0
C9—C8—H8A	109.2	C19—C20—H20	120.0
C7—C8—H8B	109.2	C20—C21—C16	121.0 (3)
C9—C8—H8B	109.2	C20—C21—H21	119.5
H8A—C8—H8B	107.9	C16—C21—H21	119.5
C8—C9—C10	121.8 (2)		
			
C10—C1—C2—C3	178.0 (2)	C2—C1—C10—C14	1.5 (4)
C5—C1—C2—C3	−1.5 (3)	C5—C1—C10—C9	8.9 (5)
C1—C2—C3—C4	26.5 (3)	C2—C1—C10—C9	−170.6 (3)
C2—C3—C4—O3	−158.4 (2)	C8—C9—C10—C1	−39.4 (4)
C2—C3—C4—C15	80.0 (3)	C8—C9—C10—C14	147.9 (3)
C2—C3—C4—C5	−40.6 (3)	C8—C7—C11—C13	−48.2 (4)
C10—C1—C5—C6	31.2 (4)	C6—C7—C11—C13	−169.0 (3)
C2—C1—C5—C6	−149.3 (2)	C8—C7—C11—C12	138.5 (2)
C10—C1—C5—C4	157.0 (3)	C6—C7—C11—C12	17.7 (3)
C2—C1—C5—C4	−23.5 (3)	C6—O2—C12—O1	173.0 (2)
O3—C4—C5—C6	−79.2 (3)	C6—O2—C12—C11	−9.5 (3)
C15—C4—C5—C6	44.9 (3)	C13—C11—C12—O1	−3.0 (4)
C3—C4—C5—C6	164.5 (2)	C7—C11—C12—O1	171.3 (3)
O3—C4—C5—C1	155.6 (2)	C13—C11—C12—O2	179.7 (2)
C15—C4—C5—C1	−80.3 (2)	C7—C11—C12—O2	−6.0 (3)
C3—C4—C5—C1	39.3 (2)	C12—C11—C13—C16	164.5 (2)
C12—O2—C6—C5	145.0 (2)	C7—C11—C13—C16	−8.2 (5)
C12—O2—C6—C7	21.0 (3)	C11—C13—C16—C17	148.9 (3)
C1—C5—C6—O2	171.08 (19)	C11—C13—C16—C21	−40.2 (4)
C4—C5—C6—O2	51.4 (3)	C21—C16—C17—C18	−1.1 (4)
C1—C5—C6—C7	−70.1 (3)	C13—C16—C17—C18	170.0 (3)
C4—C5—C6—C7	170.2 (2)	C21—C16—C17—Br1	178.06 (18)
O2—C6—C7—C11	−22.9 (2)	C13—C16—C17—Br1	−10.8 (3)
C5—C6—C7—C11	−143.8 (2)	C16—C17—C18—C19	−0.7 (4)
O2—C6—C7—C8	−149.8 (2)	Br1—C17—C18—C19	−179.9 (2)
C5—C6—C7—C8	89.3 (3)	C17—C18—C19—C20	2.0 (4)
C11—C7—C8—C9	168.4 (2)	C18—C19—C20—C21	−1.6 (4)
C6—C7—C8—C9	−74.9 (3)	C19—C20—C21—C16	−0.3 (4)
C7—C8—C9—C10	57.4 (4)	C17—C16—C21—C20	1.6 (4)
C5—C1—C10—C14	−179.1 (2)	C13—C16—C21—C20	−169.8 (2)

### Hydrogen-bond geometry (Å, º)

**Table:**

*D*—H···*A*	*D*—H	H···*A*	*D*···*A*	*D*—H···*A*
O3—H3···O2	0.77 (4)	2.26 (4)	2.883 (3)	139 (4)
